# Correlating blood-based DNA methylation markers and prostate cancer risk in African-American men

**DOI:** 10.1371/journal.pone.0203322

**Published:** 2018-09-11

**Authors:** Emmanuel Moses-Fynn, Wei Tang, Desta Beyene, Victor Apprey, Robert Copeland, Yasmine Kanaan, Bernard Kwabi-Addo

**Affiliations:** 1 Department of Biomedical Sciences, University of Maine, Orono, Maine, United States of America; 2 Laboratory of Human Carcinogenesis, Center for Cancer Research, National Cancer Institute (NCI), National Institutes of Health (NIH), Bethesda, Maryland, United States of America; 3 Department of Microbiology, Howard University, Washington, D.C., United States of America; 4 Department of Pharmacology, Howard University, Washington, D.C., United States of America; 5 Department of Biochemistry and Molecular Biology, Howard University, Washington, D.C., United States of America; National Health Research Institutes, TAIWAN

## Abstract

The objective of this work was to investigate the clinical significance of promoter gene DNA methylation changes in whole blood from African-American (AA) men with prostate cancer (PCa). We used high throughput pyrosequencing analysis to quantify percentage DNA methylation levels in a panel of 8 genes (*RARβ*2, *TIMP*3, *SPARC*, *CDH*13, *HIN*1, *LINE*1, *CYB*5*R*2 and *DRD*2) in blood DNA obtained from PCa and non-cancerous controls cases. Correlations of methylation status and various clinicopathological features were evaluated. Six genes tested achieved significant difference in DNA methylation levels between the PCa compared to control cases (P < 0.05). The *TIMP*3 loci demonstrated significant correlation of DNA methylation with age for all cases analyzed (p < 0.05). We observed an inverse correlation between *CDH*13 methylation (p = 0.045; r = -0.21) and serum vitamin D level whereas *TIMP*3 methylation (p = 0.021; r = -0.24) and *DRD*2 methylation (p = 0.056; r = -0.201) showed inverse correlation with supplementary vitamin D in the cancer cases. We also observed a direct correlation between methylation of *RARβ*2 (p = 0.0036; r = 0.293) and *SPARC* (p = 0.0134; r = 0.20) loci with PSA level in the controls but not the cancer cases. In addition, alcohol cases significantly correlated with higher *RARβ*2 methylation (p = 0.0314) in comparison with non-alcohol cases. Furthermore, we observed an inverse correlation of *DRD*2 methylation (p = 0.0349; r = -0.343) and Gleason score. Our data suggests that promoter methylation occurred more frequently in the blood of AA PCa and is associated with various clinicopathological features in AA men with PCa.

## Introduction

The incidence and mortality rate of prostate cancer (PCa) is higher for African-American (AA) men than any other US racial and ethnic groups [1,2]. It is estimated that AA men are diagnosed with PCa at approximately 60% higher rate and die from the disease at two to three fold higher rates when compared with European-American (EA) men, with AA men experiencing among the highest rates of PCa worldwide [3]. Supporting reports from autopsy data suggests higher incidence of high grade prostate intraepithelial neoplasia and PCa in AA men when compared to age-adjusted EA men. In addition, several reports have found that AA patients exhibited greater tumor volumes in comparison to similarly staged EA patients [4,5]. The disparity of PCa incidence and mortality rates is believed to be a complex integration of socioeconomic factors, environment and biology [6]. Environmental factors including dietary choice particularly the greater consumption of meat [7] and fat intake among AA men [8] may potentially contribute to increased risk of PCa and the more aggressive nature of the disease seen in AA men. While some of the PCa disparity can be attributed to environmental and/or socioeconomic factors, a number of studies points to a higher mortality rate for AA men with PCa even after adjustment for these factors [6]. Thus biological differences may also account for a significant proportion of PCa disparity in AA men in comparison to EA men [6].

The pathogenesis of PCa, like most malignancies arises from progressive acquisition of aberrant alterations in both genetic and epigenetic events. Genetic studies over the past four decades have demonstrated myriad genetic defects, including gene mutations, deletions, translocations, and amplifications, which endow the normal prostatic epithelial cells with new biological capabilities known as hallmarks of cancer [9,10]. The growing list of genetic alterations associated with PCa suggests that there is not a single predominant genetic pathway associated with the disease etiology and progression. Recent genomic advances have identified several markers that correlate with aggressive phenotypes in PCa. The TMPRSS2-ERG fusion result in androgen-regulated over-expression of ERG is thought to play a critical role in PCa [11]. While the TMPRSS2-ERG fusion is found at high frequency in EA men with PCa (~ 50%); the frequency of TMPRSS2-ERG fusion is much lower in AA men with PCa, reported to be less than 30% [11,12]. This observation suggests that there is a high frequency of the ERG-negative phenotype in AA men. Unfortunately, few studies have been carried out that compares genetic markers and their association with PCa etiology and/or progression by race and ethnicity. In addition to aberrant genetic alterations, PCa cells also carry epigenetic defects such as abnormal methylation and chromatin structure/organization patterns, with biological consequences similar to genetic changes in affecting and maintaining neoplastic and malignant phenotypes [13].

For PCa, there is abundant evidence to suggest that somatic epigenetic DNA methylation appear earlier and more frequent than other gene defects suggesting that CpG hypermethylation may be particularly important in the disease progression [14]. Like most cancers, detection of PCa at an early stage is key for the successful treatment and improvement of outcome because there is an inverse correlation between survival rates and prostate tumor aggression, despite advances in clinical surveillance and treatment strategies [15]. The level of prostate-specific antigen (PSA) in blood was commonly used for diagnosis, monitoring and prognosis. However, the era of PSA test has passed because it is not particularly specific or sensitive to detect PCa [15], therefore novel approaches to PCa screening are necessary. Thus, in addition to conventional clinicopathological features, molecular markers are needed, in particular, those associated with the malignant behavior of PCa, in order to provide the most accurate diagnostic and prognostic information. We have reported differential DNA methylation changes in benign prostate and PCa tissues for AA and European-American (EA) men using either gene specific promoter loci [16] or genome-wide DNA methylation analysis [17]. Biological consequences of aberrant DNA methylation is associated with inappropriate changes in gene expression [18] suggesting that differential methylation changes in AA and EA men could lead to differences in tumor biology and tumor aggressiveness among the two patient groups. DNA methylation changes is not only detectable in tumor samples but also in various body fluids such as blood, serum and plasma [19]. Thus, in the present study, we are interested to explore DNA methylation as non-invasive markers for PCa detection in AA men and assess their correlation with prostate cancer risk factors. Therefore, we assessed the correlation between quantitative DNA methylation changes in a panel of hypermethylated gene-specific promoters in whole blood samples and various clinical characteristics in PCa cases and controls study of patients who are self-identified as AA men.

## Materials and methods

Sample characteristics- Study population has previously been described [20,21]. Briefly, patient samples are made up of 91 African American men (cases) over the age of 40 who were diagnosed with adenocarcinoma of the prostate, and who had PSA > 3.5 ng/ml and a positive digital rectal exam (DRE). Men currently undergoing chemotherapy, radiation therapy, or androgen deprivation therapy were excluded. In addition, 91 African American men were recruited as controls from among men participating in HUH’s free Men Take Prostate Cancer Screening Program [22]. Control patients had no diagnosis of prostate cancer, PSA level < 3.5 ng/ml, negative DRE, no family history of prostate cancer among first-degree relatives, and no relationship to cases. Eligible participants were recruited from the Howard University (HU) Hospital Urology Department or from ongoing free prostate cancer screening program at the HU Cancer Center under the HU Institutional Review Board and Army Surgeon General’s Human Subject Research Review Board (HSRRB) approved the study protocol (IRB-16-MED-20). Written informed consent was obtained from all study participants. Participants completed a questionnaire to provide information on demographic (sex, marital status, income, race/ethnicity), lifestyle (smoking history, alcohol use, diet, physical activity) and occupational factors (employment status, job industry) as well as personal and family medical history (ever been diagnosed with prostate cancer, Gleason grade, PSA). A peripheral whole blood sample was collected from each participant using vacutainers containing EDTA anti-coagulant, and genomic DNA was isolated using the PUREGENE genomic DNA purification kit (Gentra Systems, Minneapolis, MN) according to the manufacturer’s protocol. Blood samples were held at room temperature (20–25°C) and were processed as soon as possible.

Quantitative DNA methylation analysis- High molecular weight genomic DNA extracted from whole blood was modified using sodium bisulfite treatment [23]. Briefly, genomic DNA (2 μg) was denatured in 0.3 mol/L NaOH at 37°C for 15 minutes; sodium bisulfite and hydroquinone were added to final concentrations of 3.1 mol/L and 0.5 mmol/L, respectively. The reaction was incubated at 50°C for 16 hours and desalted using Wizard DNA purification resin (Promega) according to the instruction of the manufacturer. Bisulfite modification was completed by DNA desulfonation in 0.3 mol/L NaOH at 37°C for 15 minutes. Modified DNA was precipitated with ethanol, washed in 70% ethanol, dried, and dissolved in 50 μL of TE buffer. The PCR primers were designed to assay the methylation status of CpGs within 0.5 kb from the transcription start site. The CpG islands interrogated were previously described; *RARβ*2, *TIMP3* and *SPARC* [16], *CDH*13, *HIN*1 and *LINE1* [24], *CYB5R2* [25] and the *DRD2* gene, a newly designed CpG island assay. Oligonucleotide sequences corresponding to PCR primers and sequencing primers are listed in the SI table. Either one-step or two-step PCR reactions were carried out using 2 μL of bisulfite-converted genomic DNA and either one or two sets of different bisulfite PCR primers in a standard PCR reaction mix. One of the primers (reverse primer) in the final PCR reaction was biotinylated to create an ssDNA template for the pyrosequencing reaction. Where indicated, we used a previously described amplification protocol [26] based on the universal primer approach. Briefly, the biotinylated reverse primer was substituted with a 5′ tailed unlabeled reverse primer and a biotinylated universal primer at a ratio of 1:9 in the PCR reaction. The integrity of the PCR product was verified on 1.5% agarose gels with ethidium bromide staining. The PCR product was immobilized on streptavidin-Sepharose beads (Amersham), washed, and denatured, and the biotinylated strands were released into annealing buffer containing the sequencing primer. Pyrosequencing was done using the PSQ HS96 Gold SNP Reagents on a PSQ 96HS machine (Qiagen). Bisulfite-converted DNA from blood of normal volunteers and blank reactions, with water substituted for DNA, served as negative control and bisulfite-converted *Sss*I methylase—treated blood DNA served as a positive control. Each bisulfite PCR and pyrosequencing reaction were done in duplicate.

### Statistical analysis

The methylation index at each gene promoter and for each sample was calculated as the average value of mC/ (mC + C; mC is methylated cytosine and C is unmethylated cytosine) for all examined CpG sites in the gene and expressed as the percentage of methylation. Z scores for DNA methylation levels of each gene were calculated across all cancer and non-cancerous control samples. Statistical significance was judged by either Mann-Whitney *t* test or Pearson correlation. The Mann-Whitney t test was used to compare DNA methylation changes in PCa cases and non-cancerous control cases. The Pearson correlation test was used to determine the correlation between DNA methylation changes versus vitamin D status/PSA/Alcohol/tobacco/age in both the PCa cases and non-cancerous control cases. The R software was used for the Mann-Whitney and Pearson correlation analysis. Standard logistic regression was used to estimate odds ratios (ORs) with 95% confidence intervals (CIs) whilst adjusting for the effects of potential confounding factors (candidate DNA methylated gene/vitamin D/alcohol/smoke) or the random association of clinicopathological variables in PCa cases relative to non-cancerous control cases. Data analysis was done using SPSS for Windows (version 18.0, SPSS) for the logistic regression. Significance was set at *P* < 0.05.

## Results

### Correlation between demographic and lifestyle characteristics and prostate cancer risk

Baseline characteristics and lifestyle behaviors for 91 control individuals and 91 PCa cases are summarized in Table 1. The mean age is 58.6 (± 9.558) for controls and 68.5 (± 9.09) for cases, indicating that the average age of cases were higher than control; and an age over 60 years was significantly associated with PCa risk when compared with the control individuals (OR = 8.06; 95% Cl = 2.44–26.58, p = 0.0006). Under the random effects model, the pooled odd ratios for the following were also observed: significant correlation of PSA and PCa risk (<10ng/ml; OR = 5.52, 95% Cl = 2.07–14.70, p = 0.0006; > 10ng/ml; OR = 27.62, 95% Cl = 3.56–213.96, p = 0.0015) for cases when compared to control individuals. The AA PCa cases who smoked increased their risk of PCa by 43% (OR = 1.43, 95% Cl = 0.774–2.65, p = 0.252) compared to control individuals; whereas AA cases who used alcohol appeared to reduce their risk to PCa by 62% (OR = 0.38; 95% Cl = 0.145–1.00, p = 0.051) when compared to non-cancerous control cases. In contrast, AA cases who were currently not drinking when compared to never drinkers only reduced their risk to PCa by 42% (OR = 0.656; 95% Cl = 0.31–1.38, p = 0.342), although this was not significant. While we do not have data on the amount of alcohol intake or the type of alcoholic beverage consumed in our patient population a recent study indicates that consumption of wine may be protective against prostate cancer [27]. African-American patients who had a family history of PCa increased their risk of getting the disease by 52% (OR = 1.52; 95% Cl = 0.758–3.04, p = 0.24), whereas a family history of benign prostatic hyperplasia significantly increased their risk to PCa by more than 2-fold (OR = 2.52; 95% Cl = 1.35–4.72, p = 0.0039). Individuals who took vitamin D supplements had a lower risk of PCa, with a significant correlation observed for individuals who took ≥400mg of vitamin D supplement (< 400mg: OR = 0.463; 95% Cl = 0.198–1.08, p = 0.075; ≥ 400mg: OR = 0.596; 95% Cl = 0.301–1.179, p = 0.0137). Furthermore, individuals with serum vitamin D level of >30ng/ml had a significant reduced risk of PCa by 30% (OR = 0.703; 95% Cl = 0.386–1.279, p = 0.025).

**Table 1:** Association between demographic and lifestyle characteristics and prostate cancer risk in African-American men.

	Control	Cases	OR, 95% CI
**Age (years)**			
<50	18	4	1.0
50–59	35	19	2.44 (0.72–8.26)
60–70	24	43	8.06 (2.44–26.58)
>70	15	25	7.70 (2.13–26.34)
**Gleason score**			
<6		39	
>7		22	
**PSA level (ng/ml)**			
≤4	58	42	1.0
<10	6	24	5.52 (2.07–14.70)
≥10	1	20	27.62 (3.56–213.96)
**Smoking status**			
Yes	55	63	1.43 (0.774–2.65)
No	35	28	
Missing	1	0	
**Alcohol consumption**			
Yes	67	55	0.38 (0.145–1.00)
Not currently	16	20	0.583 (0.192–1.774)
No, never	7	15	1.0
Missing	1	1	
**Family history of prostate cancer**			
Yes	18	25	1.52 (1.32–4.72)
No	69	63	
Missing	4	3	
**Benign Prostatic Hyperplasia**			
Yes	25	43	2.52 (1.35–4.72)
No	63	43	
Missing	3	5	
**Supplementary vitamin D (mg)**			
0	44	58	1.0
<400	18	11	0.463 (0.198–1.08)
≥400	28	22	0.596 (0.301–1.179)
**Serum vitamin D (mg/ml)**			
≤30	51	58	1.0
>30	40	32	0.703 (0.386–1.279)

A- African-American; PSA- Prostate Specific Antigen; OR- odds ratio; Cl- confidence interval.

### DNA methylation changes and prostate cancer risk in blood

In this study, we assessed the promoter methylation status in blood DNA samples extracted from 91 control and 91 PCa cases from AA men to assess the prevalence of DNA methylation changes in these 2 groups. We analyzed a total panel of 8 genes: *RARβ*2, *TIMP*3, *SPARC*, and *CYB*5*R*2 that we have previously demonstrated to be most differentially hypermethylated in PCa in AA and EA men [16] and *LINE*1, *HIN*1 and *CDH*13 that we have also shown to be most differential hypermethylated in Breast cancers from AA and EA women [24]. We have included in this study a new methylated assay of *DRD*2 promoter region, which showed hypermethylation in prostate cancer cells compared to benign primary epithelial cells. These genes were selected based on their potential role in carcinogenesis or PCa. All genes are representatives of a variety of cellular pathways that are involved in cancer, including genes that are involved in neurotransmission, cell cycle control, DNA damage response and apoptosis. The methylation status of the 8-gene panel in the PCa patients of cases and controls was further analyzed for correlation with known clinicopathologic characteristics of prostate cancer, including age of diagnosis, family history of cancer, Gleason score and life style factors such as tobacco smoke and alcohol use.

**Fig 1:**
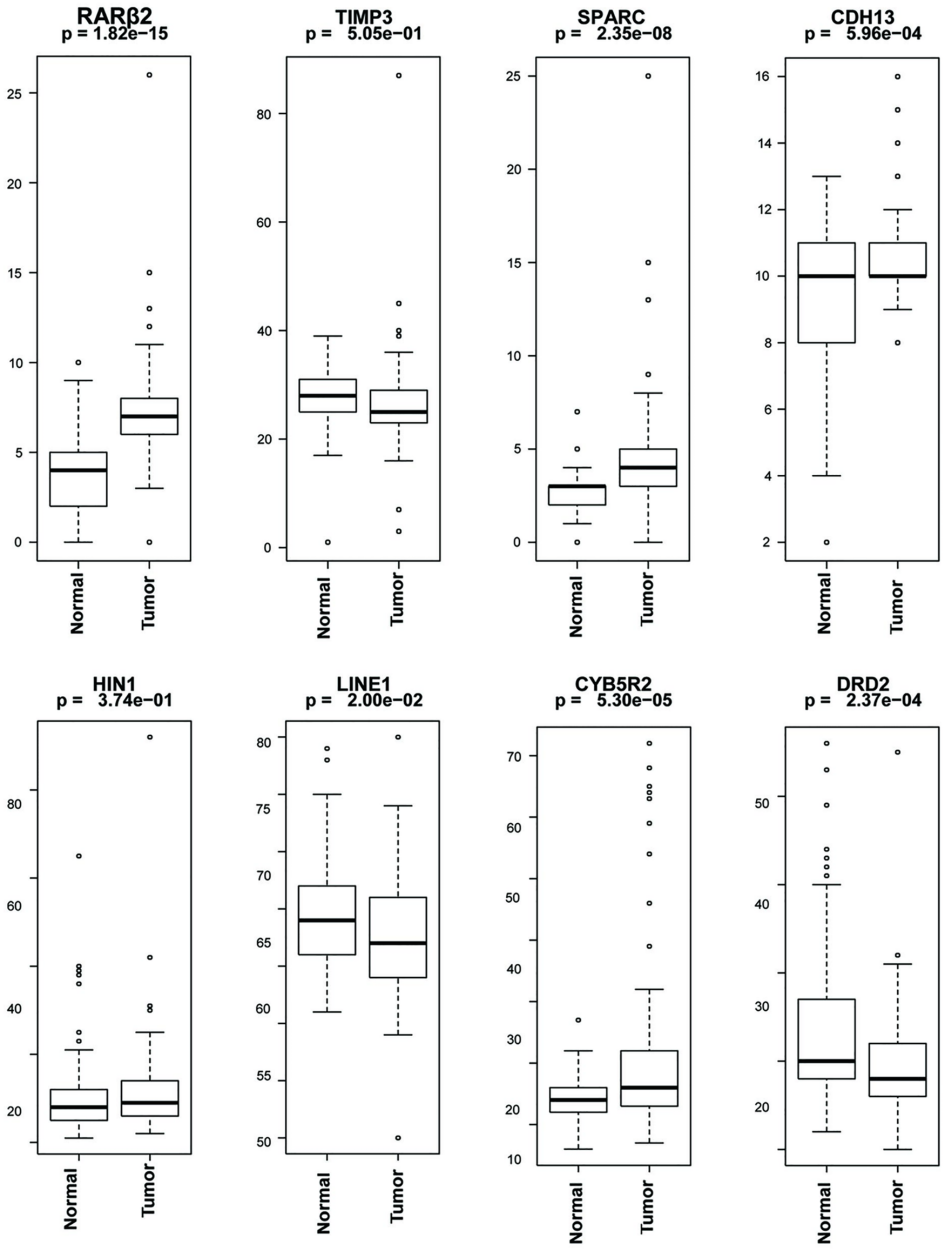
Quantitative DNA methylation analysis in blood from prostate cancer (91 cases) and non-cancerous control (91 cases). The percentage of DNA methylation levels at promoter CpG Island were analyzed in bisulfite-modified genomic DNA extracted from blood of non-cancerous (normal) and prostate cancer patient samples obtained from AA patients. *Y* axis, percentage of methylated cytosines in the samples as obtained from pyrosequencing. *X* axis, normal and tumor samples obtained from AA patients. *P* value is indicated for each gene (Mann-Whitney).

We used pyrosequencing assays to analyze the methylation status of these genes in 91 cases and 91 control cases. For each gene investigated, the percentage (%) of methylation at the specific gene promoter locus was compared between the cancer and control cases (Fig 1). Four of the genes (*RARβ*2, *SPARC*, *CDH*13, *CYB*5*R*2) showed significantly higher methylation in the cancer cases when compared with the control cases (p<0.05). On the other hand, *DRD*2 showed significantly higher methylation in the control cases when compared to the tumor cases (p = 0.0002). The *LINE*1 repetitive element, a global methylation marker was also found to have a significant reduction of methylation level in the cancer cases when compared to the control cases (p = 0.02) as previous observed by us and other [24,28].

**Fig 2:**
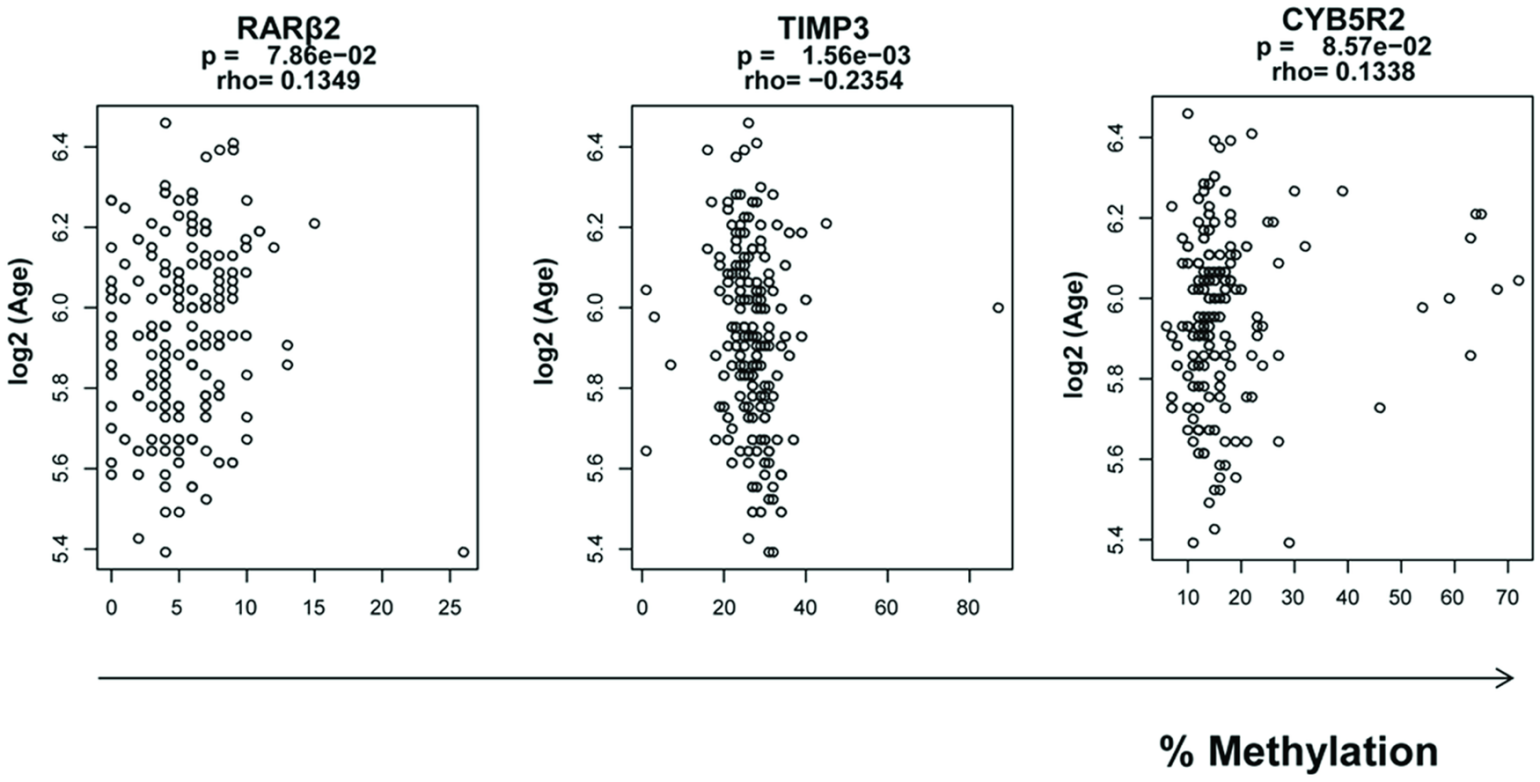
Age-related methylation analysis in blood samples from AA men. The DNA methylation level for CpG promoter sites was quantified for *RARβ*2, *TIMP*3, and *CYB*5*R*2 in 91 bisulfite-modified genomic DNA extracted from blood samples from non-cancerous AA men (age range, 42–88 y old). *Y*-axis; the log 2 value of Age (years). *X*-axis is the percentages of methylated cytosines in the samples as obtained from pyrosequencing.

To explore the correlation of the blood DNA methylation levels and patient characteristics such as age, vitamin D status, alcohol and tobacco status, we initially examined the methylation status as a function of age. We observed significant and inverse correlation between DNA methylation changes as a function of age for *TIMP*3 (p = 0.0015; r = -0.235), and a modest increase in DNA methylation change as a function of age for *RARβ*2 (p = 0.078; r = 0.134) and *CYB*5*R*2 (p = 0.086; r = 0.133; Fig 2).

**Fig 3:**
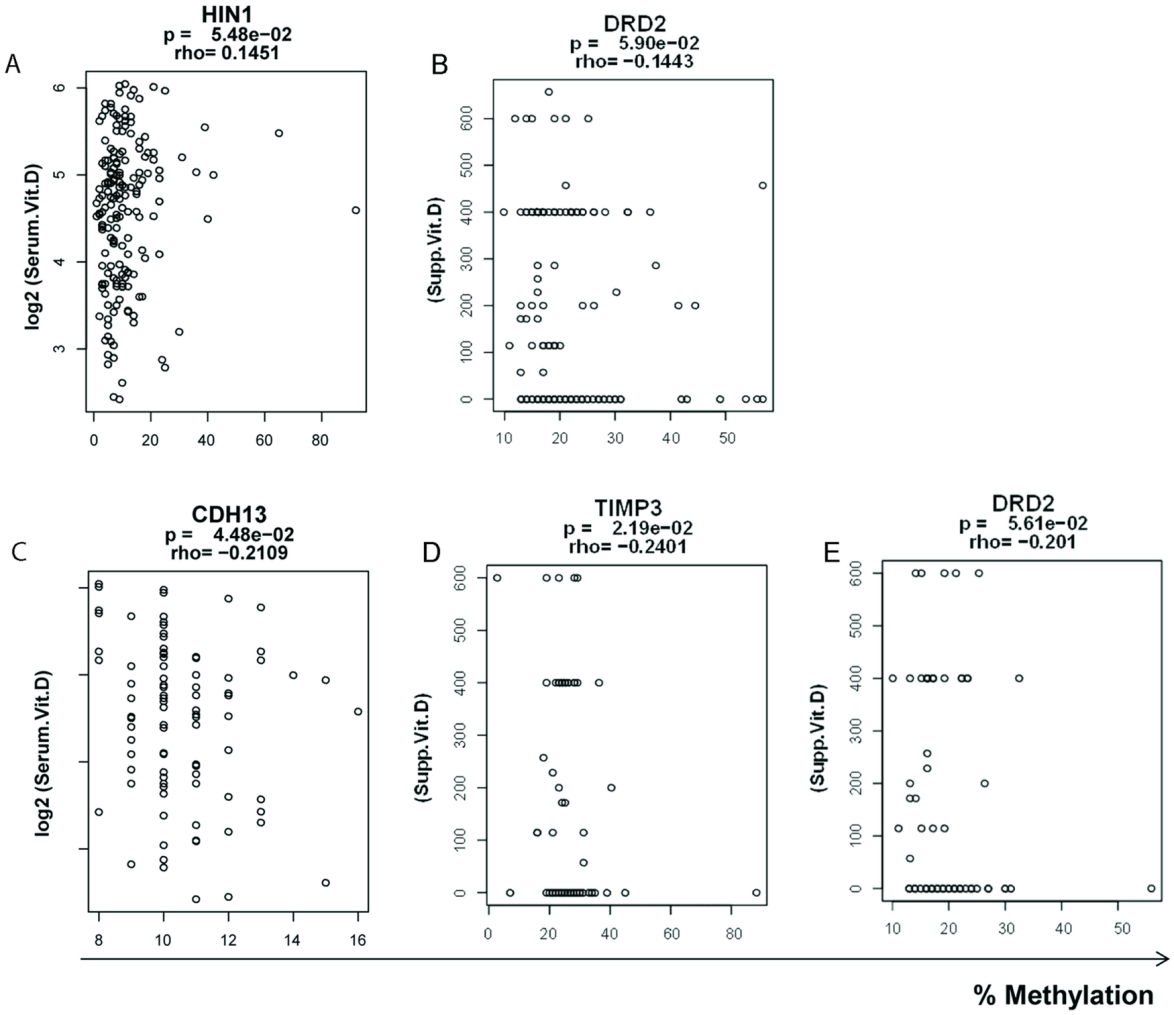
Vitamin D level and DNA methylation changes. The DNA methylation level for *HIN*1, *DRD*2, *CDH*13, and *TIMP*3 in 91 Prostate Cancer cases and 91 non-cancerous control cases were compared with either serum vitamin D level or supplementary vitamin D levels. **A**. Correlation of *HIN*1 methylation level and serum vitamin D level in the control samples. **B**. Correlation of *DRD*2 methylation and supplementary vitamin D levels in the control cases. **C**. Correlation of *CDH*13 methylation and serum vitamin D levels in cancer cases. **D**. Correlation of TIMP3 methylation and supplementary vitamin D levels in cancer cases. **E**. Correlation of DRD2 methylation and supplementary vitamin D levels. Y-axis; the log2 value for serum vitamin D or supplementary vitamin D values. X-axis is the percentage of methylated cytosines in the samples as obtained from pyrosequencing.

Next, we analyzed DNA methylation changes and vitamin D status in our patient population. We observed a significant and direct correlation of serum vitamin D and *HIN*1 DNA methylation levels (p = 0.054; r = 0.145; Fig 3A) and a modest correlation of DNA methylation of *DRD*2 (p = 0.059; r = -0.144; Fig 3B) and supplementary vitamin D level in the control cases. In the cancer cases, we observed significant and inverse correlation of serum vitamin D levels and DNA methylation of *CDH*13 (p = 0.045; r = -0.210; Fig 3C) and significant and inverse correlation of supplementary vitamin D for *TIMP*3 (p = 0.022; r = -0.24; Fig 3D) and *DRD*2 (p = 0.056; r = -0.201; Fig 3E) promoter DNA methylation.

**Fig 4:**
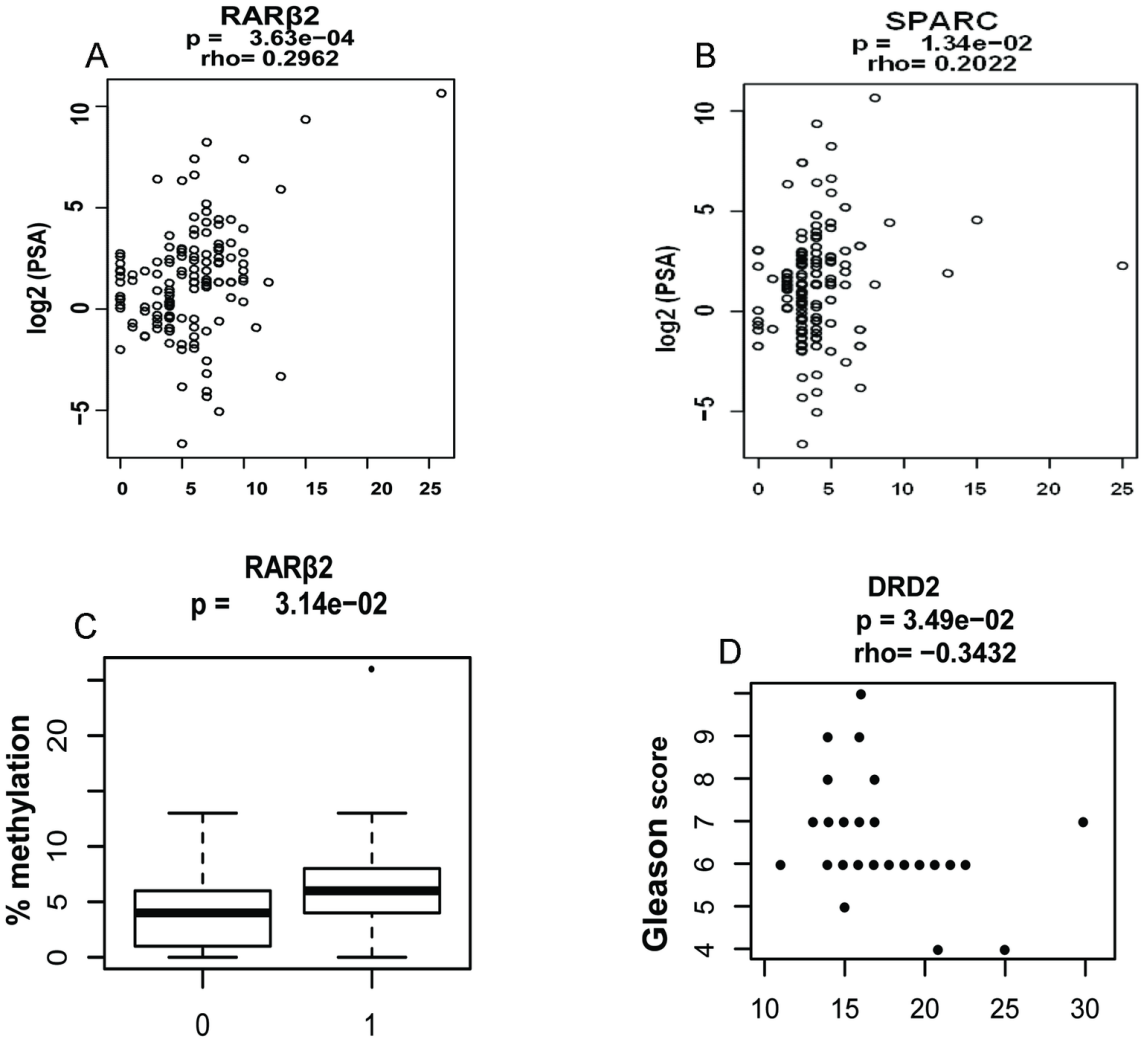
Correlation of clinocopathological data and DNA methylation changes. **A**. Correlation of PSA levels and *RARβ*2 methylation in non-cancerous control cases. **B**. Correlation of PSA levels and SPARC methylation in non-cancerous control cases. **C**. Comparison of *RARβ*2 DNA methylation changes in alcohol users (1) and never-alcohol users (0). **D**. Correlation of Gleason score and *DRD* methylation level.

We also examined the relation between PSA levels in both the control and cancer cases and DNA methylation changes and PCa Gleason score. We found significant and direct correlation of PSA and *RARβ*2 methylation (p = 0.00036; r = 0.296; Fig 4A) and *SPARC* methylation (p = 0.0134; r = 0.202; Fig 4B) in the non-cancerous control but not the cancer cases. Because tobacco and alcohol use may affect DNA methylation levels [29], we conducted a subgroup analysis comparing the methylation status for individual genes and correlation between tobacco smoke or alcohol use in cases versus control samples. We found significantly higher DNA methylation for *RARβ*2 (p = 0.0314; Fig 4C) in the non-cancerous control patients who use alcohol compared to those who never used alcohol, whereas we did not find any significant differences in DNA methylation changes in PCa patients who smoke or used alcohol compared to non-smokers or alcohol users. We also observed a significant and inverse correlation between *DRD*2 methylation (p = 0.035; r = -0.343; Fig 4D) and Gleason score. Logistic regression models were used to investigate the correlation of all genes with PCa risk while controlling for variables such as serum vitamin D, alcohol and/or smoking (Table 2). When all genes were analyzed together, we observed significant correlation of *RARβ*2 and *SPARC* methylation with increased risk of PCa whereas methylation of *TIMP*3 and *DRD*2 were significantly associated with reduced risk of PCa. When the analysis was controlled for serum vitamin D, this did not impact the correlation of *RARβ*2, *TIMP*3 and *SPARC* methylation and PCa risk except *DRD*2 methylation was longer correlated with PCa protection and *CYB*5*R*2 methylation was significantly associated with PCa risk. In addition, when the analysis was controlled for tobacco smoke or alcohol use alone, the risk of *RARβ*2 and *SPARC* methylation with PCa was higher when compared to the absence of alcohol or tobacco smoke, whereas *DRD*2 methylation was protective against PCa risk. Furthermore, the combined use of alcohol and tobacco smoke showed similar correlation effects between DNA methylation and PCa risk. However, the presence of serum vitamin D did not significantly alter the effects of alcohol and tobacco smoke on the correlation between DNA methylation changes and PCa risk. Our observation indicates that the correlation between aberrant DNA methylation levels and PCa risk can be influenced by environmental factors, however, a large population based study is required in order to validate this observation.

**Table 2:** Standard logistic analysis in prostate cancer.

	Model 1		Model 2		Model 3		Model 4		Model 5	
	Est	P value	Est	P value	Est	P value	Est	P value	Est	P value
**RARβ2**	0.61	0.0004	0.61	0.0005	0.74	0.0016	0.72	0.001	0.74	0.001
**TIMP3**	-0.21	0.028	-0.208	0.028	-0.11	0.28	-0.121	0.24	-0.121	0.25
**SPARC**	0.971	0.01	0.97	0.0105	1.49	0.006	1.40	0.007	1.35	0.009
**CDH13**	0.05	0.81	0.051	0.812	0.005	0.98	-0.004	0.98	0.022	0.92
**HIN1**	-0.0018	0.965	-0.002	0.963	0.003	0.96	0.0005	0.99	-0.003	0.95
**LINE1**	-0.100	0.251	-0.10	0.252	-0.21	0.092	-0.199	0.099	-0.19	0.12
**CYB5R2**	0.072	0.393	0.072	0.0396	0.144	0.343	0.124	0.402	0.118	0.42
**DRD2**	-0.21	0.027	0.0004	0.028	-0.264	0.054	-0.25	0.053	-0.244	0.059
**SVD**	n/a	n/a	0.0004	0.973	n/a	n/a	n/a	n/a	0.008	0.66
**Smoke**	n/a	n/a	n/a	n/a	1.46	0.184	n/a	n/a	n/a	n/a
**Alcohol**	n/a	n/a	n/a	n/a	2.32	0.096	n/a	n/a	n/a	n/a
**Smoke + Alcohol**	n/a	n/a	n/a	n/a	n/a	n/a	1.74	0.09	1.74	0.96

Logistic regression model to determine the correlation of DNA methylation level and prostate cancer risk while adjusting for confounding factors or life-style factor (serum vitamin D levels; tobacco smoke; alcohol consumption). **Model 1**: Correlation of all genes and prostate cancer risk. **Model 2**: Correlation of all genes and prostate cancer risk adjusting or controlling for serum vitamin D level. **Model 3**: Correlation of all genes and prostate cancer risk adjusting or controlling for either tobacco smoke or alcohol consumption. **Model 4**: Correlation of all genes and prostate cancer risk adjusting or controlling for both tobacco smoke and alcohol consumption. **Model 5**: Correlation of all genes and prostate cancer risk adjusting or controlling for both tobacco and alcohol consumption and serum vitamin D level. Est: Estimate (exponential of the estimate gives odds ratio); n/a (non-applicable). P- Value of statistical significance; p <0.05.

**Fig 5:**
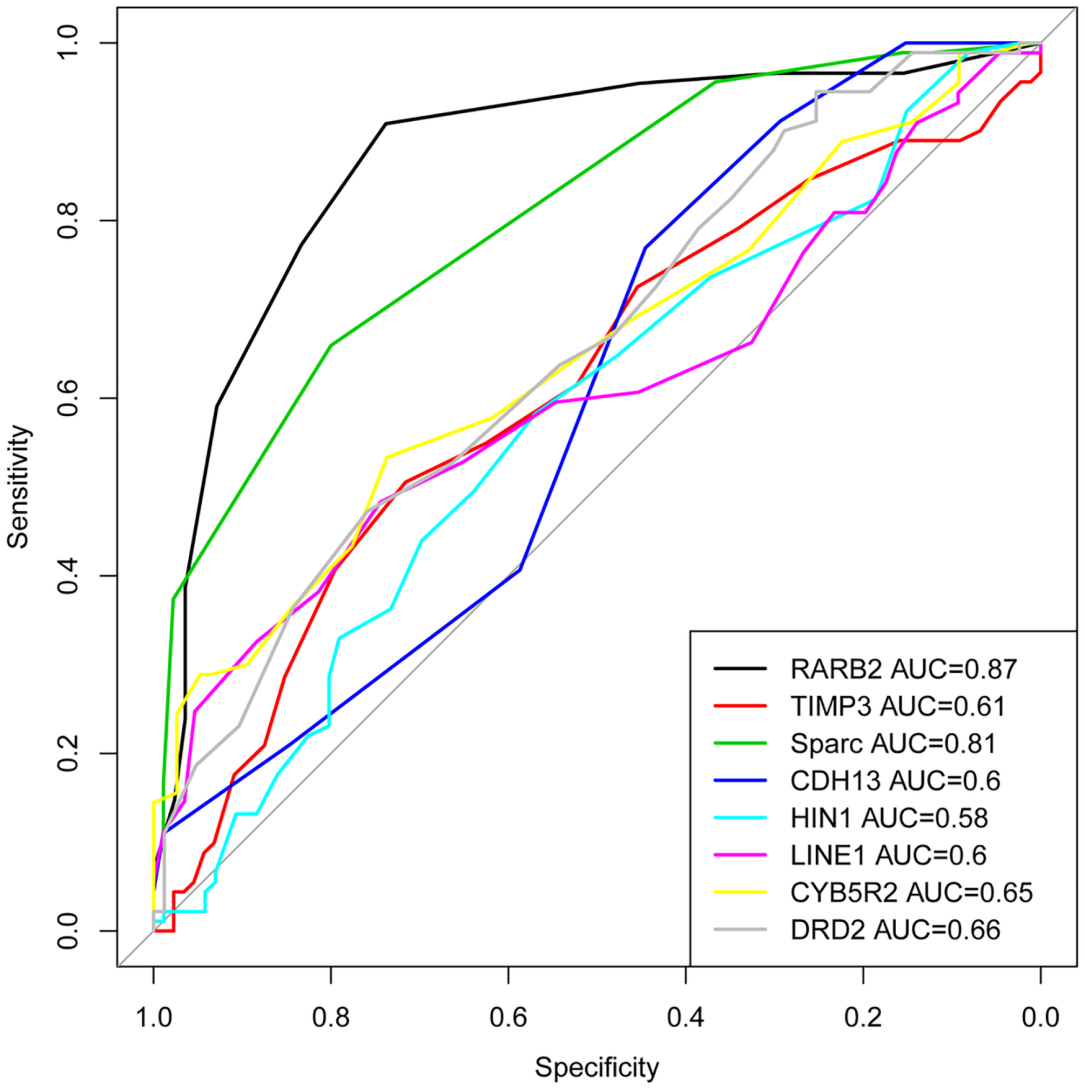
AUC curves for methylation analysis in blood samples from AA PCa cases. The areas under the curves in blood samples are 0.87 for *RARβ*2, 0.81 for *SPARC*, 0.66 for *DRD*2, 0.62 for *CYB*5*R*2, 0.61 for *TIMP*3, 0.60 for *CDH*13, 0.60 for *LINE*1 and 0.58 for *HIN*1.

Finally, we used area under curves (AUCs) analysis (Fig 5) to compare DNA methylation levels in cancer versus non-cancerous control cases. The AUCs were as follows: 0.87 for *RARβ*2, 0.81 for *SPARC*, 0.66 for *DRD*2, 0.62 for *CYB*5*R*2, 0.61 for *TIMP*3, 0.60 for *CDH*13, 0.60 for *LINE*1 and 0.58 for *HIN*1. The strength of the AUCs indicates differences in the sensitivity and specificity of different DNA methylated genes for PCa detection in blood with the highest sensitivity observed for *RARβ*2 and *SPARC* genes although the sensitivity is somewhat lower than we had previously observed in PCa tissues [16]. Although the sample size used in our studies is not large, the AUCs analysis suggests the potential use of DNA methylated genes for PCa detection in blood.

## Discussion

There is a need to improve existing method for PCa detection and screening and to distinguish indolent from aggressive disease. Cancer patients typically have higher levels of cell free DNA in comparison to individuals without cancer and genomic DNA originates from cancer cells has been routinely detected in clinical specimens (e.g. plasma/serum, sputum, urine etc) [30]. Epigenetic DNA methylation precede clinically obvious cancer and is more common and consistent than genetic alterations [31], and has the potential as biomarkers for PCa detection, disease prognosis and treatment [32]. DNA methylation is ubiquitous in several body fluids and has the potential for detecting the presence of cancer in plasma/serum or urine and thereby eliminates the harm associated with biopsy [33]. A July 31, 2018 PubMed search for “prostate cancer etiology and biomarkers and DNA methylated genes and blood” identified 11 articles describing DNA methylated genes in PCa including promoter methylation of the *GSTP*1 (glutathione S-transferase P1) gene which is the most common methylated gene reported thus far for PCa, appearing earlier and more frequently than other gene defects that arise during PCa development [31,34]. Hypermethylation of *GSTP*1 is detected in 12% of all clinically localized PCa and in 28% of hormone refractory metastatic PCa [35]. In addition, hypermethylation of *GSTP*1 is a strong predictor for PSA recurrence following radical prostatectomy [35]. However, most studies exploring the biomarker potential of DNA methylated genes (including *GSTP*1) have focused on samples from EA individuals with few studies carried out of samples from African-American, a population that is disproportionally affected by PCa. Previous reports have shown that different organs showed tissue-specific methylation patterns [36,37], we have demonstrated differential methylation in prostate and breast tissues from AA and EA men and women respectively [16,24] thus the plasticity, tissue-specific nature, and variability of methylated pattern across individuals suggesting that studies based on different populations are needed to provide more insights into the fundamental dynamics of DNA methylation changes in different clinical specimens.

In this study, we aimed to identify the association of DNA methylation changes and PCa risk factors in whole blood samples from AA men. To our knowledge, this is the first study to explore DNA methylation changes and PCa risk factors in blood samples from AA men. The results of our analysis showed that methylation of *RARβ*2, *SPARC*, *CDH*13, *LINE*1, *CYB*5*R*2 and *DRD*2 promoter gene loci was significantly associated with PCa risk in AA men. Aberrant methylation was detected not only in patients with PCa but also in some of the control cases. One report identified aberrant methylation of several genes in non-cancerous breast tissues and blood DNA of smokers [38] suggesting that the presence of aberrant methylation in blood DNA could predict early events of neoplastic transformation, exposure (a biologic effect of an environmental toxicant) or still unidentified environmental carcinogenic factors. In this study, 5 out of 8 genes investigated demonstrated significant higher DNA methylation in the blood from PCa cases when compared to the non-cancerous control cases. This result provides strong, albeit indirect, evidence that the methylated DNA of the specified gene originates from the cell free DNA of prostate cancer and is not affected by the blood DNA from other non-malignant tissues. The lower methylation of *LINE*1 in the blood from PCa cases relative to the control cases is consistent with previous report of hypomethylation of *LINE*1 in blood DNA associated with increased risk to bladder cancer [39] suggesting that the methylation status may not be influenced by blood DNA from non-cancerous tissues. We have previously reported higher DNA methylation of *TIMP*3 gene in PCa tissues in comparison with non-cancerous prostate tissues from both AA and EA men [16]. However, in the present study we observed lower *TIMP*3 methylation in blood from the cancer cases in comparison with the non-cancerous cases albeit insignificant suggesting that *TIMP*3 methylation in blood may be influenced by some environmental factors as previously proposed [40] but not investigated in our present study. We also observed lower methylation frequency for *DRD*2 gene in blood from PCa cases in comparison with the control cases. In addition, the *DRD*2 methylation in the blood samples from PCa cases showed an inverse correlation with vitamin D status and tobacco smoke as well as alcohol use suggesting that *DRD*2 methylation was directly influenced by these dietary and lifestyle factors. We also observed significant correlation between *DRD*2 methylation levels and Gleason score. The *DRD*2 methylation may be directly influenced by environmental factors such as vitamin D levels as well as tobacco and alcohol use, lifestyle factors that are linked to PCa risk (mentioned above) and could therefore be a marker for PCa aggression. In support of this observation one report has demonstrated that polymorphisms in *DRD*2 were associated with tobacco smoke in PCa patients [41].

There may be a role for vitamin D in PCa etiology and progression as high incidence of PCa in AA men is linked to deficiency of vitamin D [42]. Some reports suggest an inverse correlation of the intensity and duration of sunlight exposure with PCa which may be explained by a decreased vitamin D synthesis [43], whereas others report small to no effect of vitamin D and PCa risk [44], thus epidemiologic studies have reported inconsistent association of vitamin D and PCa risk. We observed significant correlation of aberrant methylation for *CDH*13, *HIN*1, *TIMP*3 and *DRD*2 genes in blood vitamin D level. Our results showed that both serum vitamin D and supplementary vitamin D were associated with reduced PCa risk in AA PCa patients suggesting that if vitamin D is playing a role in biology of PCa, it may be using epigenetic mechanisms such as DNA methylation. Additional large population study is required which includes history of dietary pattern and other lifestyle choices in order to identify the risk factors associated with methylation and tumor progression.

Hypermethylation of *RARβ*2 and correlation with PSA levels in PCa patients has previously been reported [45]. In this study, we observed significant correlation of PSA levels and DNA methylation changes for *RARβ*2 and *SPARC* loci in the non-cancerous control samples to suggest that the basis of the aberrant methylation maybe influenced by some environmental exposures (such as the consumption of alcohol) in our patients samples analyzed which is consistent with our observation of significantly higher RARβ2 methylation in alcohol users when compared to never-alcohol users, however we cannot rule out other factors such as dietary folate [46] that was not investigated in the present study.

Alcohol is a potential human carcinogen and studies have revealed that chronic alcohol drinking is linked to elevated homocysteine (Hcy) levels in the plasma [47] and Hcy is involved in the metabolism of methyl groups, substrate for DNA methylation. The biologic effect of alcohol may interact with other factors such as tobacco smoke (common co-occurring life-style risk factors) to induce abnormal DNA methylation changes. In our logistic regression analysis tobacco smoke and alcohol use were significantly associated with aberrant methylation of *RARβ*2, *SPARC*, *LINE*1 and *DRD*2 loci in AA patients with PCa suggesting that methylation of these genes could be associated with increased risk of PCa in patients who smoke tobacco and or consume alcohol. There are numerous reports to show that alcohol action is directly associated with neurotransmitters such as dopamine and serotonin and its receptors [48,49]. Our present observation of aberrant methylation in the *DRD*2 gene and alcohol and/or tobacco use in blood samples from AA patients with PCa indicates that the status of *DRD*2 methylation is correlated with lifestyle factors such as alcohol consumption and tobacco smoke in addition to serum vitamin D (discussed above) in AA PCa patients. Thus, our panel of methylation markers is of interest for further evaluation as serum-based biomarkers and may help identify high-risk AA individuals so that the PCa could be diagnosed at early stage. Aging is a risk factor for PCa, and DNA methylation changes are linked to mechanisms that drive aging [50]. We observed close correlation between aging and DNA methylation of *RARβ*2, *CYB*5*R*2 and *TIMP*3 suggesting that the prevalence of these methylation signals in these cancer-related genes could heighten the development of PCa in older AA men.

Strengths of our studies included the valid ascertainment of PCa cases and statistical adjustment for known and suspected PCa risk factors. Our study used pyrosequencing to measure DNA methylation levels as this is a high-throughput, quantitative method that is considered highly sensitive to detect differences in DNA methylation between individuals. In addition, the large, prospective design of the study and our ability to separately evaluate the correlation between blood DNA methylation measures and prostate cancer risk was a further strength. Limitation includes our study population of only AA men and may not be generalizable to other populations. We also acknowledge that tumor contamination is an important factor to consider as DNA methylation levels in blood may not reflect levels in prostate tissue. We did not have available data in our study for DNA methylation in the prostate tissues for comparison. However, several reports have demonstrated correlation of DNA methylation changes in tumor and blood samples suggesting the potential utility of blood DNA methylation as independent predictors of cancer risk for a variety of solid tumors [51], including for example the *GSTP*I and prostate cancer [52]. Our small sample size has limited power to detect most gene-gene and/or gene-environment interactions and prostate pathogenesis however, our finding of significant correlation of *RARβ*2 methylation with increased PCa risk for the interaction of age, vitamin D status and alcohol and tobacco smoke suggests a potential molecular mechanism of prostate pathogenesis that results from aberrant methylation patterns due to environmental exposures. The *RARβ2* methylation could serve as a potential biomarker for early diagnosis of PCa in AA men.

In summary, we have found aberrant DNA methylation of 6 (out of a panel of 8) genes in blood that correlates with PCa risk and may serve as indicators of environmental or lifestyle factors that biologically contributes to PCa risk in AA men through “epigenome-environment” interaction. Results from this study suggest the need for large scale follow-up blood-based monitoring of this panel of gene methylation to examine their role in AA PCa and the disease disparity.

## Supporting information

S1 Table Information of oligonucleotide primer sequences used in PCR and pyrosequencing analysis. (DOCX)
