# Supplementary material for: Correlating blood-based DNA methylation markers and prostate cancer risk in African-American men
Source: PLoS One. 2018 Sep 11;13(9):e0203322. doi: 10.1371/journal.pone.0203322 (PMC6133349; doi:10.1371/journal.pone.0203322)
Supplement: S1 Table — (DOCX) [file pone.0203322.s001.docx]

|  | Pyrosequencing |  |  |
| --- | --- | --- | --- |
|  | **Sense** | **Antisense** | **Sequencing primer** |
| RARβ2  (1^st^ step) | AGTTGGGTTATTTGAAGGTTA | TACCCAAACAAACCCTACTC and U-CCCAAACAAACCCTACTC | GGGACACCGCTGATCGTTTA |
| RARβ2  (2^nd^ Step) | AAGTAGTAGGAAGTGAGTTGTTTAGA | 5’-Biotin-U |  |
| TIMP3  (1^st^ Step) | GGTGGGTGGGTGTTAGTTGG | U-CAAACCCTCCTACCCCTTCTC | GGTTAGAGATATTTAGTGGTTTA |
| TIMP3  (2^nd^ Step) | GGTTTTGGTTTGGGTTAGAGATA | 5’-Biotin-U |  |
| SPARC  (1^st^ Step) | GGTGTAATTATAGAAGGGAAAGGTTGGG | U-CCTATTACCTATCTCTAAACCCCTCCACATT | TTAGGGTAGTTTGAAGGAT |
| SPARC  (2^nd^ Step) | AAGGTTGGGAGGGGGTTATATATATTTTAG | 5’-Biotin-U |  |
| CDH13 (1^st^ Step) | TTTGGGAAGTTGGTTGGTTG | ACAACCCCTCTTCCCTACCT | AGGAAAATATGTTTAGTGTA |
| CDH13 (2^nd^ Step) | AGTTTGGTTTTTAAGGAAAATATGTTTAGT | Biotin-AACCAAATTCTCCACTACATTTTATCC |  |
| HIN1  (1^ST^ Step) | GGGGAGTTTATAGGAGTTGTAGGATAG | AACCAAACCAACAAAACTTTCTCAA | GGGTTAAGTAGAGTTTTAGGAG |
| HIN1  (2^ND^ Step) | GGGGAGTTTATAGGAGTTGTAGGATAG | U-ACCAAAACCCAATATAAAAAACCT  5’-Biotin-U |  |
| LINE1 | TTTTGAGTTAGGTGTGGGATATA | Biotin-AAAATCAAAAAATTCCCTTTC | AGTTAGGTGTGGGATATAGT |
| CYB5R2 | GGTTTTGGGTTAGTTTTGTTTTTAGG | Biotin-ACCTTCTCCAACCTTACC | GGTTAGTTTTGTTTTTAGGG |
| DRD2 | TTAGGTAAGGAGAGGAGGTGTT | Biotin- ACTAAAATCCAAAACCTTAAATACATCA | AGGAGGTGTTGGAAG |

**Table S.** Primer sequences used in the pyrosequencing analysis.

U represents universal primer sequence- GGGACACCGCTGATCGTTTA
